# BRD4 inhibitor GNE987 exerts anti-cancer effects by targeting super-enhancers in neuroblastoma

**DOI:** 10.1186/s13578-022-00769-8

**Published:** 2022-03-18

**Authors:** Yan-Ling Chen, Xiao-Lu Li, Gen Li, Yan-Fang Tao, Ran Zhuo, Hai-Bo Cao, Wan-yan Jiao, Zhi-Heng Li, Zhen-Hong Zhu, Fang Fang, Yi Xie, Xin-Mei Liao, Di Wu, Hai-Rong Wang, Juan-Juan Yu, Si-Qi Jia, Yang Yang, Chen-Xi Feng, Peng-Cheng Yang, Xiao-Dong Fei, Jian-Wei Wang, Yun-Yun Xu, Guang-Hui Qian, Zi-Mu Zhang, Jian Pan

**Affiliations:** 1 Institute of Pediatric Research, Children’s Hospital of Soochow University, No. 92 Zhongnan Street, SIP, Suzhou, 215003 China; 2 School of Basic Medicine and Biological Sciences, Soochow University, Suzhou, 215003 China; 3 Burn and Plastic Surgery, Children’s Hospital of Soochow University, Suzhou, 215003 China; 4 Radiology Department, Children’s Hospital of Soochow University, Suzhou, 215003 China

**Keywords:** BRD4, Neuroblastoma, Broad H3K27ac domain, Super-enhancer, PROTAC

## Abstract

**Background:**

Neuroblastoma (NB) is a common extracranial malignancy with high mortality in children. Recently, super-enhancers (SEs) have been reported to play a critical role in the tumorigenesis and development of NB via regulating a wide range of oncogenes Thus, the synthesis and identification of chemical inhibitors specifically targeting SEs are of great urgency for the clinical therapy of NB. This study aimed to characterize the activity of the SEs inhibitor GNE987, which targets BRD4, in NB.

**Results:**

In this study, we found that nanomolar concentrations of GNE987 markedly diminished NB cell proliferation and survival via degrading BRD4. Meanwhile, GNE987 significantly induced NB cell apoptosis and cell cycle arrest. Consistent with in vitro results, GNE987 administration (0.25 mg/kg) markedly decreased the tumor size in the xenograft model, with less toxicity, and induced similar BRD4 protein degradation to that observed in vitro. Mechanically, GNE987 led to significant downregulation of hallmark genes associated with *MYC* and the global disruption of the SEs landscape in NB cells. Moreover, a novel candidate oncogenic transcript, *FAM163A*, was identified through analysis of the RNA-seq and ChIP-seq data. *FAM163A* is abnormally transcribed by SEs, playing an important role in NB occurrence and development.

**Conclusion:**

GNE987 destroyed the abnormal transcriptional regulation of oncogenes in NB by downregulating BRD4, which could be a potential therapeutic candidate for NB.

**Supplementary Information:**

The online version contains supplementary material available at 10.1186/s13578-022-00769-8.

## Introduction

Neuroblastoma (NB) is the most common extracranial solid tumour of childhood, and is derived from primordial neural crest cells [1]. In the past 20 years, although the treatment intensity and treatment options for high-risk NB have continued to increase, the 5-year overall survival rate of high-risk NB is still less than 50%, and this condition accounts for more than 15% of childhood deaths caused by cancer [2, 3]. Clinical research has shown that the majority of patients with metastatic NB respond well to early-stage cytotoxic chemotherapy; however, patients with recurrent NB tend to exhibit drug resistance [1, 4]. To date, because of various treatment strategies, the prognosis of patients with high-risk NB has improved for decades.

Based on the hallmarks of malignant tumour phenotypes, e.g., rapid proliferation, invasion, and distal metastasis, continually active transcription is essential in tumour cells. Previous studies have proven that compared with somatic cells, cancer cells generally present an increased level of overall transcriptional output and oncogenic transcriptional activity, allowing more opportunities to participate in carcinogenic pathways [5, 6]. The transcriptional activation of target genes mainly depends on associated transcription factors and enhancers. Enhancers can activate the transcription of linear distant genes independent of their position and orientation, and their dysregulation is often associated with tumorigenesis and development [7, 8]. Super-enhancers (SEs) are large clusters of multiple enhancer-like elements, spanning several kilobases in size that can increase the transcription of their target genes compared with typical enhancers [9, 10]. Existing studies have revealed that the anomalous expression of critical oncogenes in most paediatric cancers is dependent on aberrant transcription induced by SEs, including that of *MYCN* (encoding the MYCN proto-oncogene, a BHLH transcription factor) in NB [11, 12], *MYC* (encoding the MYC proto-oncogene, a BHLH transcription factor) in T-cell acute lymphoblastic leukaemia [13], *GFI1* (encoding the growth factor independent 1 transcriptional repressor) in medulloblastoma [14], and *JUN* (encoding the Jun proto-oncogene, an AP-1 transcription factor subunit) in glioblastoma [15]. Transcriptional regulation of the above oncogenes is highly sensitive to the disruption of SEs. Thus, it would be advantageous to discover chemical inhibitors that specifically target to SEs for clinical therapy of malignant tumours.

SEs are large regulatory DNA fragments that are enriched with many additional transcription factors, chromatin regulators, and cofactors, thus, uncovering the possible intervention targets involved in the composition of SEs should be an effective strategy to inhibit SE-associated transcription [15]. Currently, the SE-related components recognized as cancer therapeutic targets are bromodomain containing 4 (*BRD4*), cyclin dependent kinase 7 (*CDK7*), E1A binding protein P300 (*EP300*), *CDK8* and *CDK19* [16]. Among them, *BRD4* [17], *CDK7* [18], and *EP300* [16] are components of the SE complex, which mediates the positive transcriptional regulation of SE-targeted genes, while *CDK8* [19] and *CDK19* [20] act as negative factors. For example, the classic SE inhibitors, JQ1 [21, 22], THZ1 [23, 24], and CBP30 [16] target *BRD4*, *CDK7*, and *EP300*, respectively, inhibiting the occupancy of *BRD4* on its targets, the phosphorylation of Pol II and the enrichment of acetylated histone H3K27 (*H3K27ac*), leading to the collapse of SEs and the substantial downregulation of SE-related oncogene transcription.

Among them, inhibitors targeting BRD4 are the most widely used. BRD4 is a member of the bromodomain and extraterminal (BET) family, which also includes BRD2, BRD3 and BRDT [25]. Mechanistically, *BRD4* specifically recognizes and binds to acetylated lysine residues of histones, recruiting the positive transcription elongation factor *P-TEFb*, and participates in the control of the transcriptional elongation of RNA-polymerase II (RNA-Pol II) to preferentially regulate SE associated genes [26–28]. To date, approximately 20 BRD4 inhibitors have been assessed in clinical trials, and some of them have exhibited superior therapeutic effects for lymphoma, multiple myeloma and non-small-cell lung cancer (statistics from Clinicaltrials.gov: https://clinicaltrials.gov/ct2/home) [29]. However, most of these molecules act by matching the binding ligand or active site and require a high concentration in vivo for a long time, which inevitably leads to off-target and side effects [30].

In contrast to traditional design methods for small molecule drugs, proteolysis targeting chimaera (PROTACs) are a novel technology. PROTACs are heterobifunctional small molecule compounds with two recruiting ligands connected via a linker, one of which is specific to the target protein and the other of which is responsible for the recruitment of an E3 ligase, committing the target protein to ubiquitination and subsequent degradation [31, 32]. With these advantages, PROTAC technology has been applied in the synthesis of BET inhibitors for the degradation of proteins [33]. As we reported previously, ARV-825 is a PROTAC-based BRD4 inhibitor that can connect BRD4 with the E3 ligase cereblon (CRBN) and exhibits outstanding antitumour effects on NB [34]. Recently, Arvinas LLC (http://ir.arvinas.com/) reported that in a phase I clinical trial of prostate cancer, ARV-110 (NCT03888612 in Clinicaltrials.gov) [a PROTAC probe targeted to the androgen receptor (AR)] showed a good therapeutic effect, with DC50 values (the concentration at which 50% of the protein is degraded) of ~ 1 nM, and also demonstrated its safety and tolerability in patients with metastatic castration-resistant prostate cancer (mCRPC) [35, 36].

GNE987 is a newly developed von Hippel-Lindau tumour suppressor (VHL)-based pan-BET-targeting PROTAC, that can bind to target proteins (BET proteins, including BRD2, BRD3, and BRD4) and recruit them to the ubiquitin/proteasome system for selective degradation [37, 38]. However, the function of GNE987 has not been assessed in NB thus far. In the present study, we aimed to examine the effect of GNE-987 on NB cell lines and xenograft mouse models in vitro and in vivo.

## Results

### High BRD4 expression is associated with poor prognosis in NB patients

**Fig. 1:**
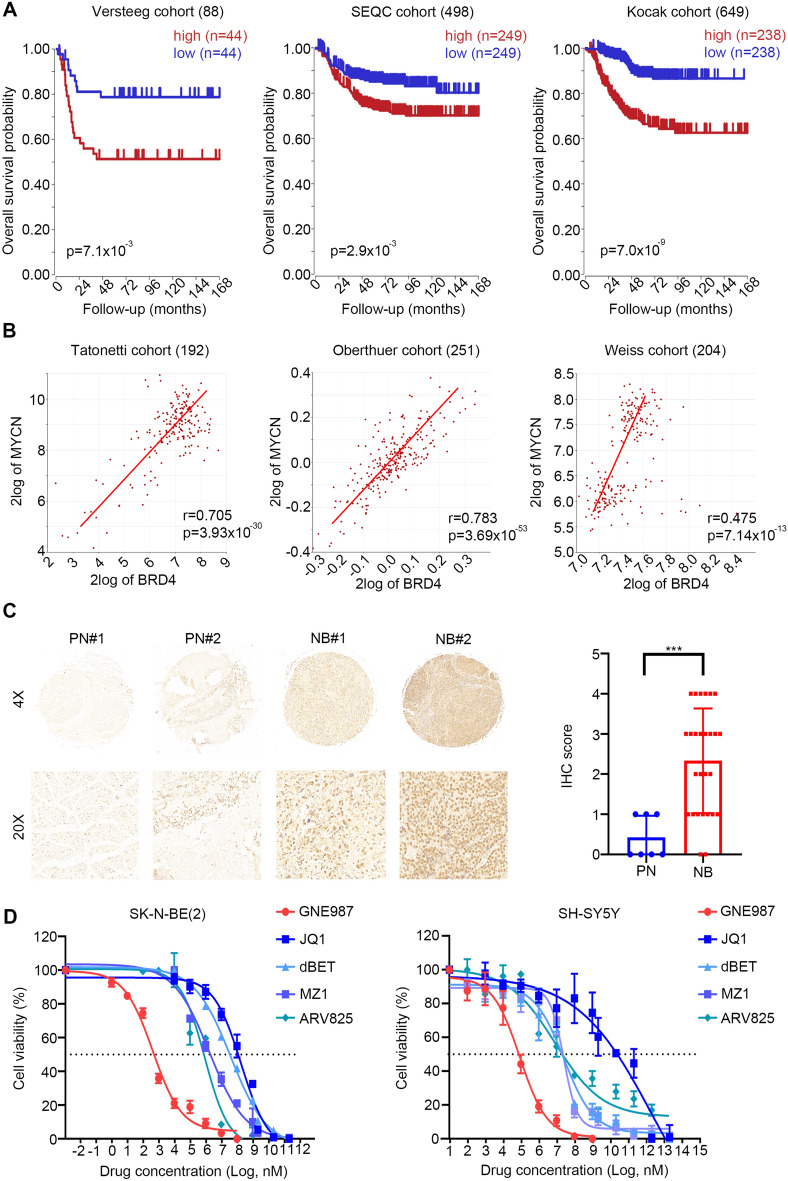
High *BRD4* expression in NB was associated with poor prognosis. **A** Kaplan–Meier analysis of the correlation of *BRD4* high or low expression with the OS of the patients with NB [generated from R2 Genomics Analysis and Visualization Platform (http://r2.amc.nl)]. Median survival time was used as a cutoff point for defining high or low expression; p < 0.001. **B** Correlation between *BRD4* and *MYCN* mRNA expression in NB [generated from R2 Genomics Analysis and Visualization Platform (http://r2.amc.nl)]; p < 0.001. **C** Representative images of IHC staining of the *BRD4* protein on a tissue microarray constructed from 27 NB tissues and seven matched normal tissues. Histological scores were determined according to the intensity of *BRD4* staining. PN, peripheral neuron; NB, neuroblastoma. ***p < 0.001. **D** Dose–response curves of five *BRD4* inhibitors (GNE987, JQ1, dBET, MZ1, ARV825) at the indicated concentrations in SK-N-BE (2) or SH-SY5Y cells. Data are represented as the mean ± SD

To determine the potential utility and clinical relevance of targeting BRD4 in NB, we evaluated the prognostic significance of *BRD4* and the correlation of its mRNA expression with *MYCN* mRNA levels in published NB patient datasets. We first analysed the association of the *BRD4* expression level with the overall survival of patients with NB using three different cohorts in the R2 platform. Based on the publicly available datasets of 88 patients (GSE16476), 498 patients (GSE49710) and 649 patients (GSE45547) derived from the GEO database, Kaplan–Meier curves were constructed. The median value of *BRD4* expression was used as a cutoff point to categorize high or low expression. Kaplan–Meier survival curve analysis showed that the patients with high *BRD4* expression had markedly shorter overall survival times than those with low *BRD4* expression (Fig. 1A). Furthermore, in 192, 251, and 204 of the NB clinical samples analysed in the GSE126575, E-TABM-38, and GSE55248 arrays from the R2 database, *BRD4* expression correlated positively with the expression of the NB tumour driver *MYCN*, indicating that high *BRD4* expression was associated with the tumorigenesis and development of NB (r = 0.705, p = 3.93 × 10^–30^; r = 0.783, p = 3.69 × 10^–53^; r = 0.475, p = 7.14 × 10^–13^) (Fig. 1B). Next, *BRD4* was examined using immunohistochemistry (IHC) on samples from 27 patients with NB and 7 corresponding peripheral nerve tissues (Fig. 1C). The results showed stronger nuclear staining of BRD4 in the NB tissues than in the peripheral nerve tissues, indicating an elevated BRD4 protein level in NB tissues. Taken together, these data indicated that ectopic expression of BRD4 contributed to the occurrence of tumour and was associated with a poorer prognosis in patients with NB, revealing that BRD4 could be an important therapeutic target in patients with NB.

Previous studies showed that other PROTAC inhibitors (JQ1, dBET, MZ1, and ARV825) also inhibited NB cell proliferation through BRD4 [34, 39]; therefore, we performed a comparison between GNE987 and the above BRD4 inhibitors to determine their different rates of inhibition of cell viability. Five groups with equal numbers of NB cells (SK-N-BE (2) or SH-SY5Y) were exposed to increasing concentrations of GNE987, JQ1, DBET, MZ1, and ARV825 for 24 h and cell viability was evaluated using CCK-8 assays. The five groups of NB cells showed various degrees of dose-dependent growth inhibition after drug treatment (Fig. 1D). However, GNE987 inhibited NB cell proliferation at the lowest concentration, with IC50 values that were at least 100-fold lower than those of the other inhibitors, suggesting a superior inhibitory effect of this novel PROTAC bromodomain inhibitor on NB cells.

### NB cells are sensitive to GNE987 treatment

**Fig. 2:**
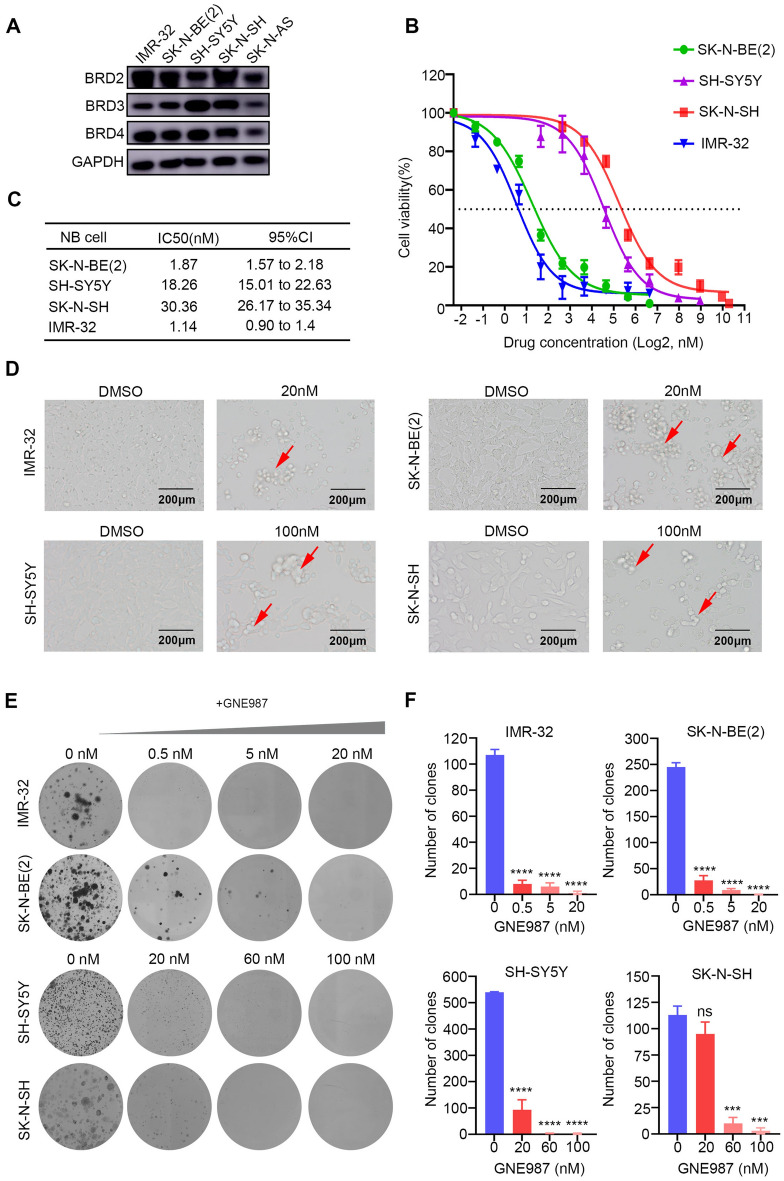
GNE987 inhibited NB cell viability and proliferation. **A** BET protein levels in NB cells were assessed using Western blotting. **B** Survival rate curves of the SK-N-BE (2), IMR-32, SK-N-SH, and SH-SY5Y cells after treatment with vehicle (DMSO) or increasing concentrations of GNE987 for 24 h. Data are shown by percent cell viability relative to that of the DMSO-treated cells. **C** The IC50 value of GNE987 in different NB cell lines. **D** Microscopic images of the SK-N-BE (2), IMR-32, SK-N-SH and SH-SY5Y cells treated with vehicle (DMSO) and the specified concentrations of GNE987 for 24 h (red arrows indicate dead cells after GNE987 treatment). **E**, **F** The clonal formation ability of the NB cells treated with the vehicle or a series of concentrations of GNE987 as assessed using a clone formation assay. ***p < 0.001, ****p < 0.0001; ns, not significant

To investigate the functional effects of GNE987 based on BRD4-targeted inhibition in NB cells, we first examined BRD4 protein levels in NB cell lines. BRD4 was abundantly expressed in both human and mouse NB cell lines, indicating that BET family members are universally expressed in NB (Fig. 2A). We then examined the cell-killing effects of the BRD4 inhibitor GNE987 on NB cell lines in vitro. Four NB cell lines were treated with the vehicle control (0.1% DMSO) or decreasing concentrations of GNE987 ranging from 5 to 0 μM for 48 h, and cell viability was measured using a CCK-8 assay. GNE987 exhibited an obvious inhibitory effect on IMR-32, SK-N-BE (2), SK-N-SH and SH-SY5Y cell lines (IMR-32 IC50, 1.14 nM; SK-N-BE (2) IC50, 1.87 nM; SK-N-SH IC50, 30.36 nM; and SH-SY5Y IC50, 18.26 nM) (Fig. 2B and C). Moreover, abnormal morphological features were observed in the GNE987 treatment group, with clustered and floating cells (Fig. 2D). Additionally, we assessed the effect of GNE987 therapy on clonogenic survival over a prolonged period of time. The results showed that GNE987 inhibited NB cell colony formation in a concentration-dependent manner compared with that in the control groups (Fig. 2E and F). Collectively, these data suggested that GNE987 exerted a strong cytotoxic effect on NB cell lines in vitro at nanomolar concentrations.

### GNE987 induces cell cycle arrest and apoptosis in NB cells

**Fig. 3:**
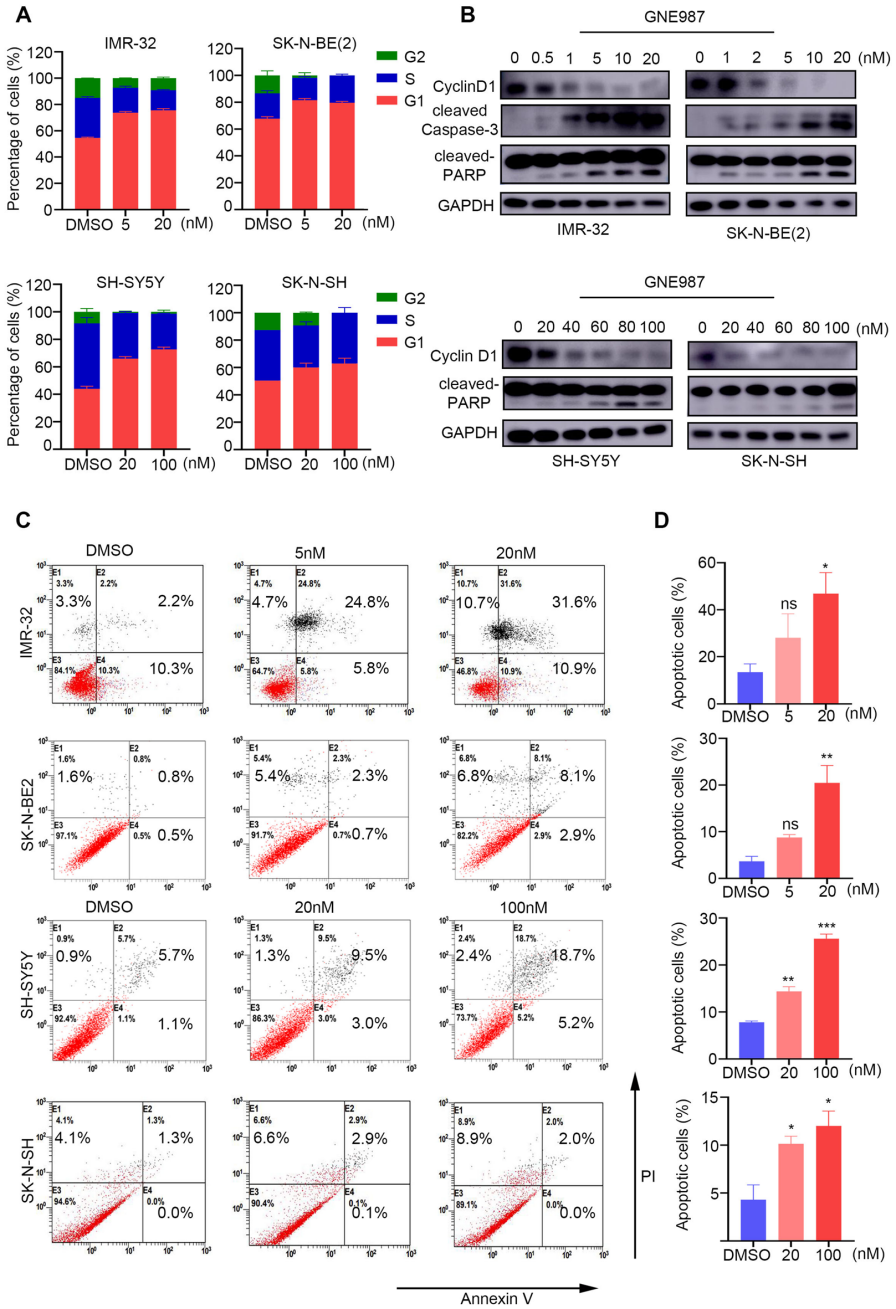
GNE987 inhibited the NB cell cycle and induced apoptosis. **A** NB cells were treated with GNE987 for 24 h and the cell cycle distribution of NB cells was analysed using flow cytometry. **B** Western blotting analysis showing the expression of *cyclin D2*, cleaved *caspase-3*, and cleaved *PARP* in the NB cells treated with vehicle (DMSO) or increasing concentrations of GNE987 for 24 h. **C**, **D** Annexin-V/PI staining and flow cytometry were performed to detect apoptosis in the NB cells treated with vehicle (DMSO) or the specified concentrations of GNE987. *p < 0.05, **p < 0.01, ***p < 0.001

Previously, BRD4 was reported as a mitotic marker and implicated in cell-cycle regulation, which was associated with the transcription start sites of M/G1 genes [40]. GNE987 directly targets BRD4; therefore, flow cytometry was performed to assess the effect of GNE987 on the cell cycle distribution in NB cell lines. The results showed that GNE987 increased G1/S phase arrest in NB cell lines compared with that of the control groups (Fig. 3A and Additional file 1: Fig. S1). Additionally, another important regulator of the cell cycle, cyclin D1, showed continuously downregulated expression with increasing concentrations of GNE987 (Fig. 3B). To further explore the effect of GNE987 on cell proliferation, we assessed cell apoptosis using Annexin V/PI staining and detected using flow cytometry (Fig. 3C). The results showed that all four NB cell lines underwent GNE987 dose-dependent apoptosis, accompanied by PARP and/or caspase-3 cleavage (Fig. 3D), supporting the hypothesis that GNE987 affects the cell cycle and induces apoptosis of NB cells by regulating *BRD4*.

### GNE987 degrades BET proteins and reduces the expression of downstream N-Myc or C-Myc proteins in NB cells

**Fig. 4:**
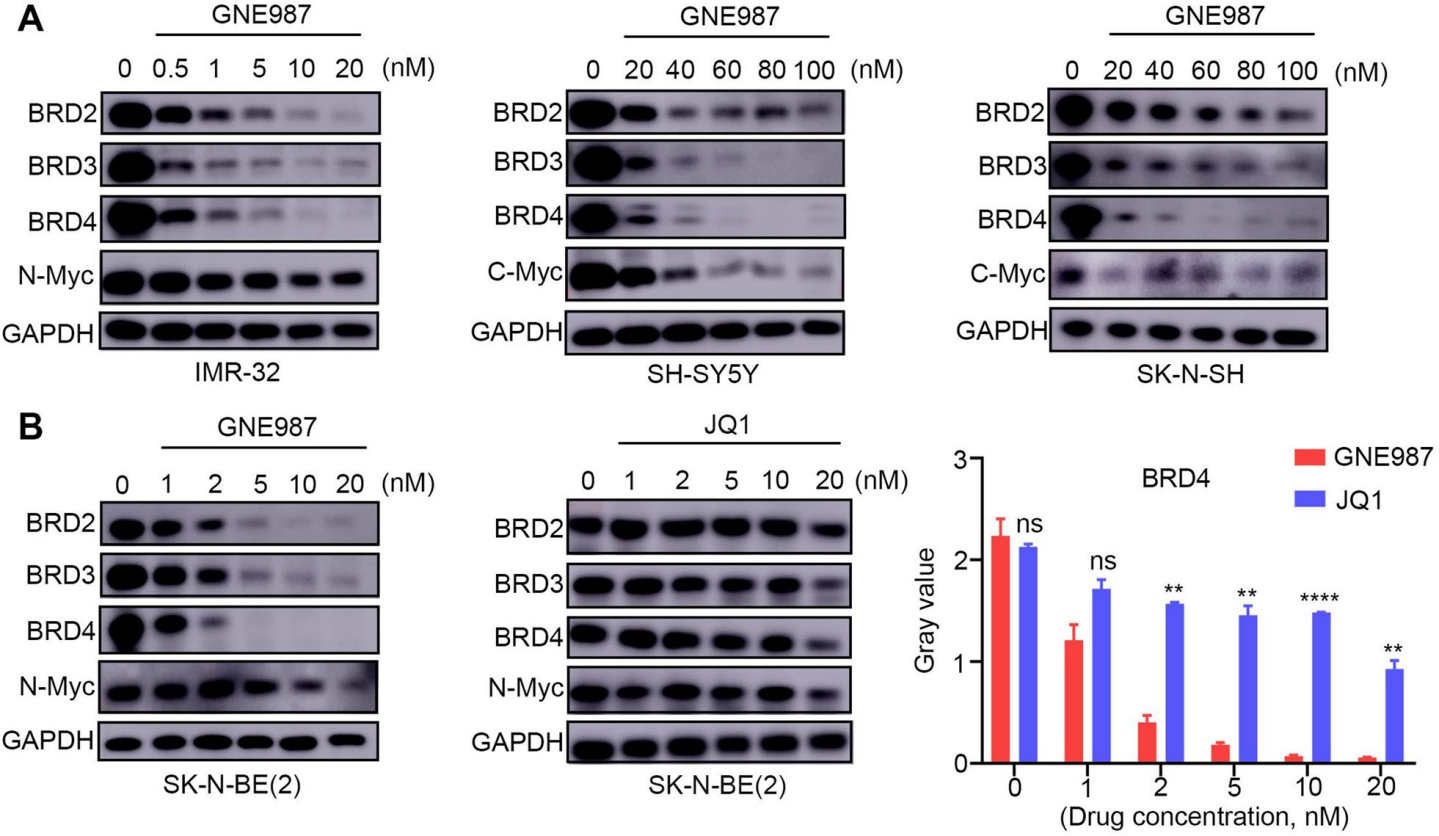
GNE987 induced the degradation of a BET protein and N-myc or C-myc. **A** Western blotting analysis of *BET* proteins and N-myc or C-myc levels in the NB cells treated with vehicle (DMSO) or. increasing doses of GNE987 for 24 h. **B** Protein levels of BET and N-myc in the SK-N-BE (2) cells treated with vehicle (DMSO), GNE987, or JQ1. The BRD4 protein level was normalized against that of *GAPDH*. *p < 0.05, **p < 0.01, ***p < 0.001, ****p < 0.0001; ns, not significant

As a new type of proteolytic targeting chimaera, GNE987 is a bifunctional molecule that can promote the degradation of protein targets. Western blotting was performed to determine the BET protein level following GNE987 treatment. The results showed that all four cell lines exhibited GNE987 concentration-dependent BET degradation activity, with a preferential degradation effect on BRD4 over BRD2 and BRD3. Next, because previous studies demonstrated that BET bromodomains are transcriptional regulators of N-Myc or C-Myc and that deregulation of BET resulted in suppression of N-Myc or C-Myc transcription [41], we further determined whether GNE987 influenced the downstream targets of BRD4. As expected, the N-Myc or C-Myc protein level decreased dramatically and dose-dependently after BET depletion by GNE987 in NB cells (Fig. 4A). In addition, under the same conditions, JQ1 treatment resulted in less downregulation of N-Myc expression and weaker degradation of BET proteins, suggesting that GNE987 has a stronger degradation effect on the BET and N-Myc proteins than JQ1 (Fig. 4B).

### VHL expression is indispensable for sensitivity to GNE987

**Fig. 5:**
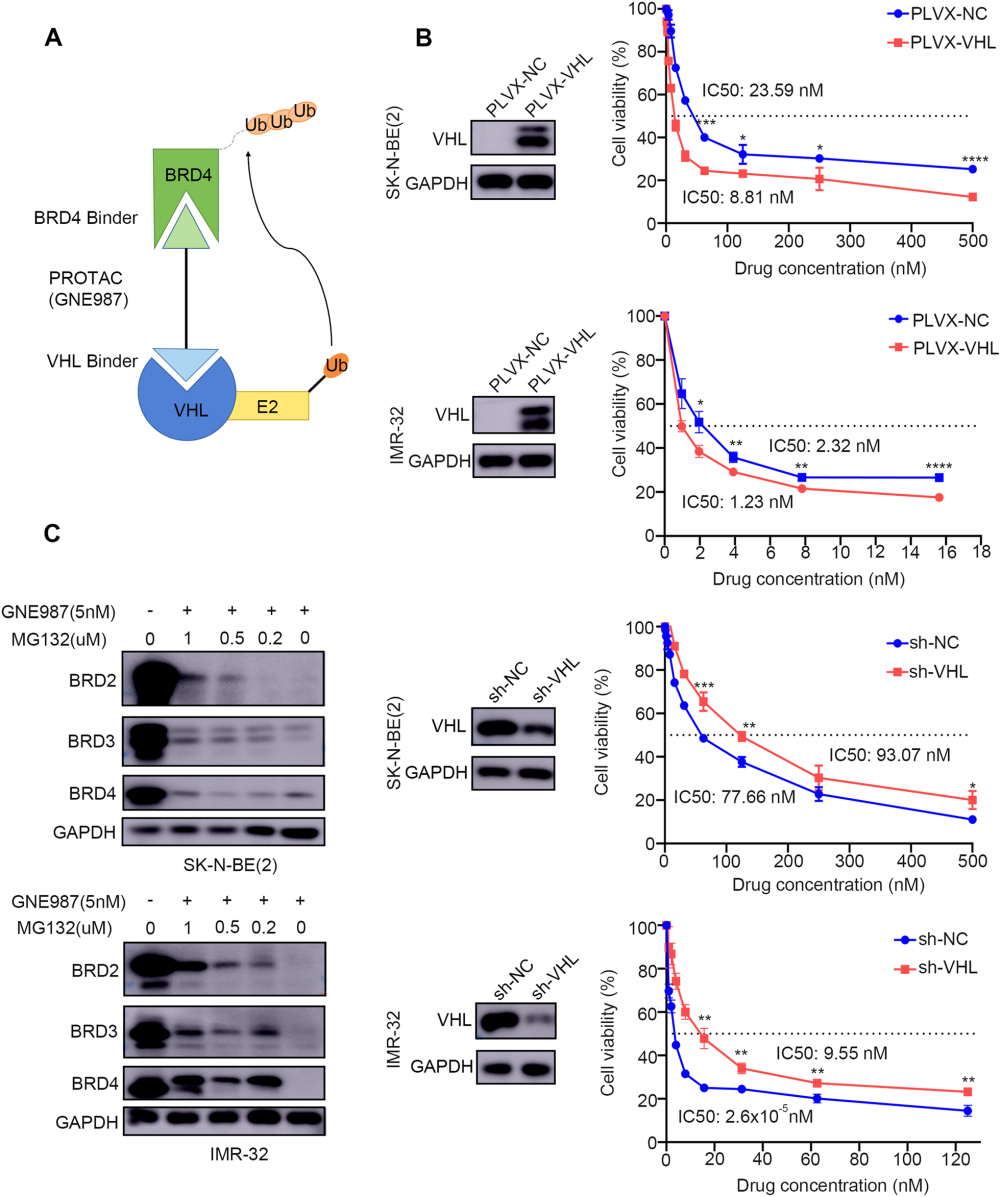
GNE987 is a *BET*-targeting PROTAC based on *VHL*, for which the *BET* protein degradation is proteasome-dependent. **A** Bifunctional PROTAC molecules bind to the targeting-protein (*BRD4*, dark green) with one end (a motif, light green) while the other end (a motif, light blue) binds to an E3-ubiquitin ligase (dark blue) to form a ternary complex. The recruited E3 ligase then mediates the transfer of ubiquitin from an E2 enzyme to the targeted protein (direction of arrow). **B** Analysis of *VHL* expression in the IMR-32 and SK-N-BE (2) cells treated with negative control sh-NC (or PLVX-NC) and sh-*VHL* (or PLVX-*VHL*) examined by Western blotting. Then the sensitivity to GNE987 of the NB cells transfected with sh-*VHL* (or PLVX-*VHL*) and NB cells transfected with sh-NC (or PLVX-NC) was compared. *p < 0.05, **p < 0.01, ***p < 0.001, ****p < 0.0001. **C** Western blotting analysis of the *BET* protein in SK-N-BE (2) and IMR-32 cells treated with the 5 nM GNE987 and series concentrations of MG132, and their combination for 24 h

Previously, it was reported that GNE987-induced degradation of BET family proteins depended on the binding of the substrate recognition subunits of VHL; therefore, we investigated the sensitivity to GNE987 of NB cell lines with low or high VHL expression levels (via overexpression or knockdown of *VHL*) (Fig. 5A). Western blotting showed overexpression of VHL in stably transfected SK-N-BE (2) and IMR-32 cell lines. A CCK-8 assay indicated that overexpression of VHL greatly increased sensitivity to GNE987 in IMR-32 and SK-N-BE (2) cells compared with those transfected with the empty vector. In contrast, after treatment with the same concentrations of GNE987, depletion of VHL using the short hairpin RNA (shRNA) in IMR-32 and SK-N-BE (2) cells increased cell survival compared with that of the cells transfected with the scrambled shRNA (sh-NC) (Fig. 5B). Given that BET proteins are rapidly converted through ubiquitin-dependent degradation and that other PROTAC bromodomain inhibitors of BET protein (including ARV825) also downregulate the expression of BET proteins through this mechanism, the reliance of GNE987-mediated BET protein degradation on ubiquitin was examined using the cell permeable proteasome inhibitor MG132 (Fig. 5C). As expected, cotreatment with MG132 greatly attenuated the ability of GNE987 to induce BET degradation. Taken together, the results indicated that VHL is essential for GNE987 to exert its antitumour effect in NB cells.

### GNE987 has a strong antitumour effect in an NB xenograft mouse model

**Fig. 6:**
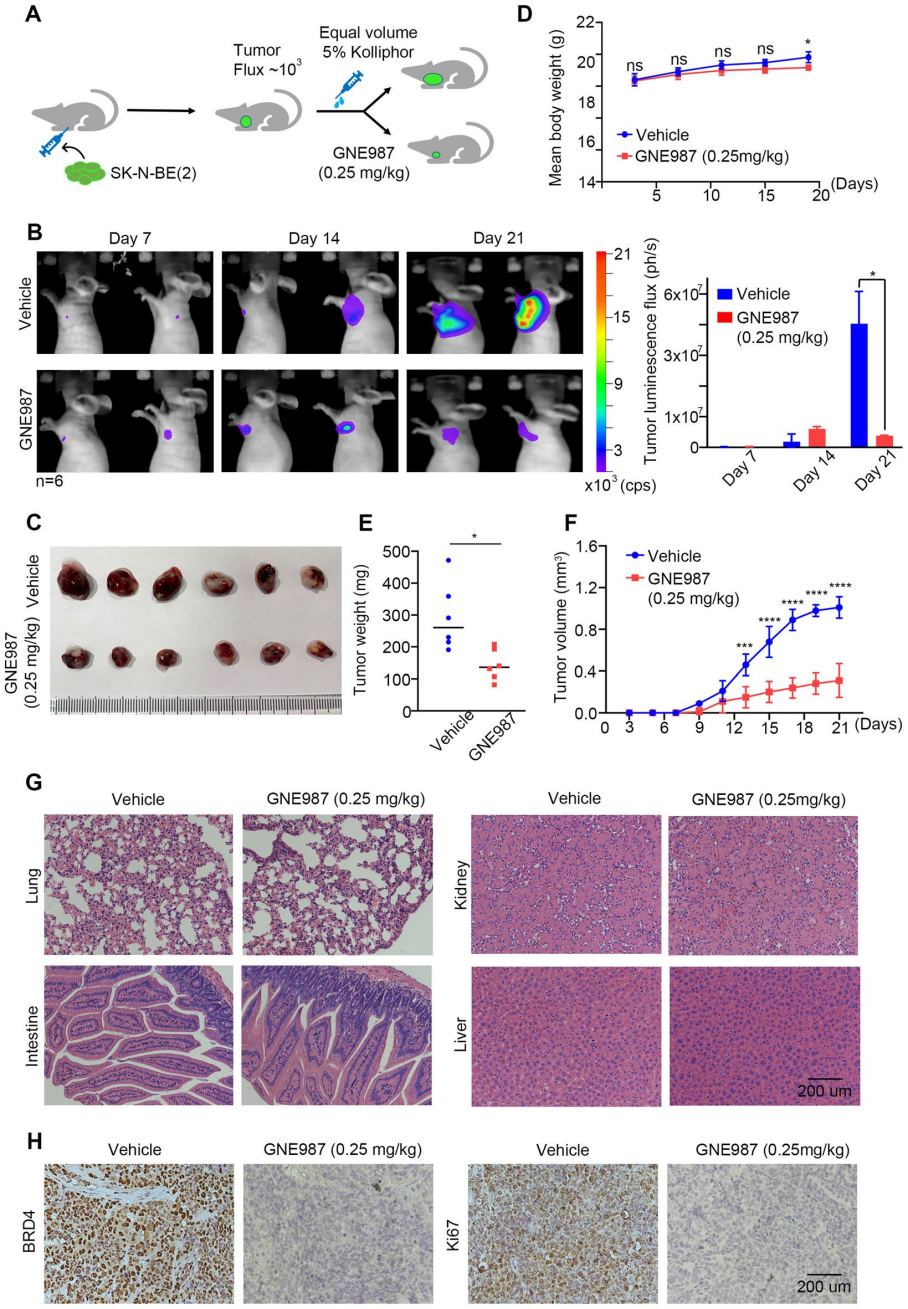
In vivo GNE987 treatment caused tumour necrosis in NB xenograft models. **A** Schematic of the experimental design. Mice bearing luciferase-labelled NB xenograft tumours were treated with intraperitoneal injections of 0.25 mg/kg GNE987 every other day once their tumour luminescence flux reached 3–5 × 10^5^ photons/second via bioluminescent imaging (BLI). **B** Representative sequential BLI images of the tumour bearing mice in the control group or the GNE987 treatment group taken at 7, 14, and 21 days after subcutaneous implantation of NB cells. Scale is in photons/second. n = 6. *p < 0.05. **C** Photograph of excised tumours from the mice in the GNE987 treatment group and the control group at d 21. Scale bar, 1 cm. **D** Weight changes in the tumour bearing mice in the GNE987 treatment group and the control group. **E** The average tumor weight of the excised tumours at d 21. **F** Tumour growth curves of tumour-bearing mice in the GNE987 treatment group and the control group in response to different treatments. ***p < 0.001, ****p < 0.0001. **G** H&E staining of organs in the mice in the GNE987 treatment group. Scale bar, 200 μm. **H** Representative images of IHC staining with the indicated antibodies in tumours harvested from the mice in the GNE987 treatment group and the control group. Scale bar, 200 μm

To further test the feasibility and efficiency of GNE987 in the treatment of NB in vivo, we established an NB model by subcutaneously xenotransplanting luciferase-labelled human NB cells [SK-N-BE (2)] into nude mice. Following initial tumour establishment, one group of tumour-carrying nude mice (n = 6) was treated with an intraperitoneal injection of 0.25 mg/kg GNE987 every other day, while the mice in the other group were injected with the vehicle at the same dosage as the test group (Fig. 6A). Tumour growth was monitored using a live animal bioluminescence imaging system every week. The results showed a significant reduction in tumour luminescence signals on the third week in the mice treated with GNE987. In contrast, tumour luminescence signals from the control group continued to rise acutely (Fig. 6B). Measurements of the final tumour luminescence signal before dissection were compared with the actual size of the tumours, which confirmed that the changes in luminescence signals accurately reflected the changes in tumour size in the mouse model (Fig. 6C). Additionally, following three weeks of GNE987 therapy, although the tumour mass and volume in the treatment group were significantly diminished compared with those in the control group, this treatment had little effect on the body weight of the mice and caused no overt toxicities (Fig. 6D–G). IHC data demonstrated that GNE987 decreased the levels of BDR4 and Ki-67 in the tumours, which was consistent with the results from the in vitro experiments (Fig. 6H). Collectively, these data suggested that GNE987 had a strong antitumour effect on NB in vivo.

### Mechanism of the antitumour effect of GNE987

**Fig. 7:**
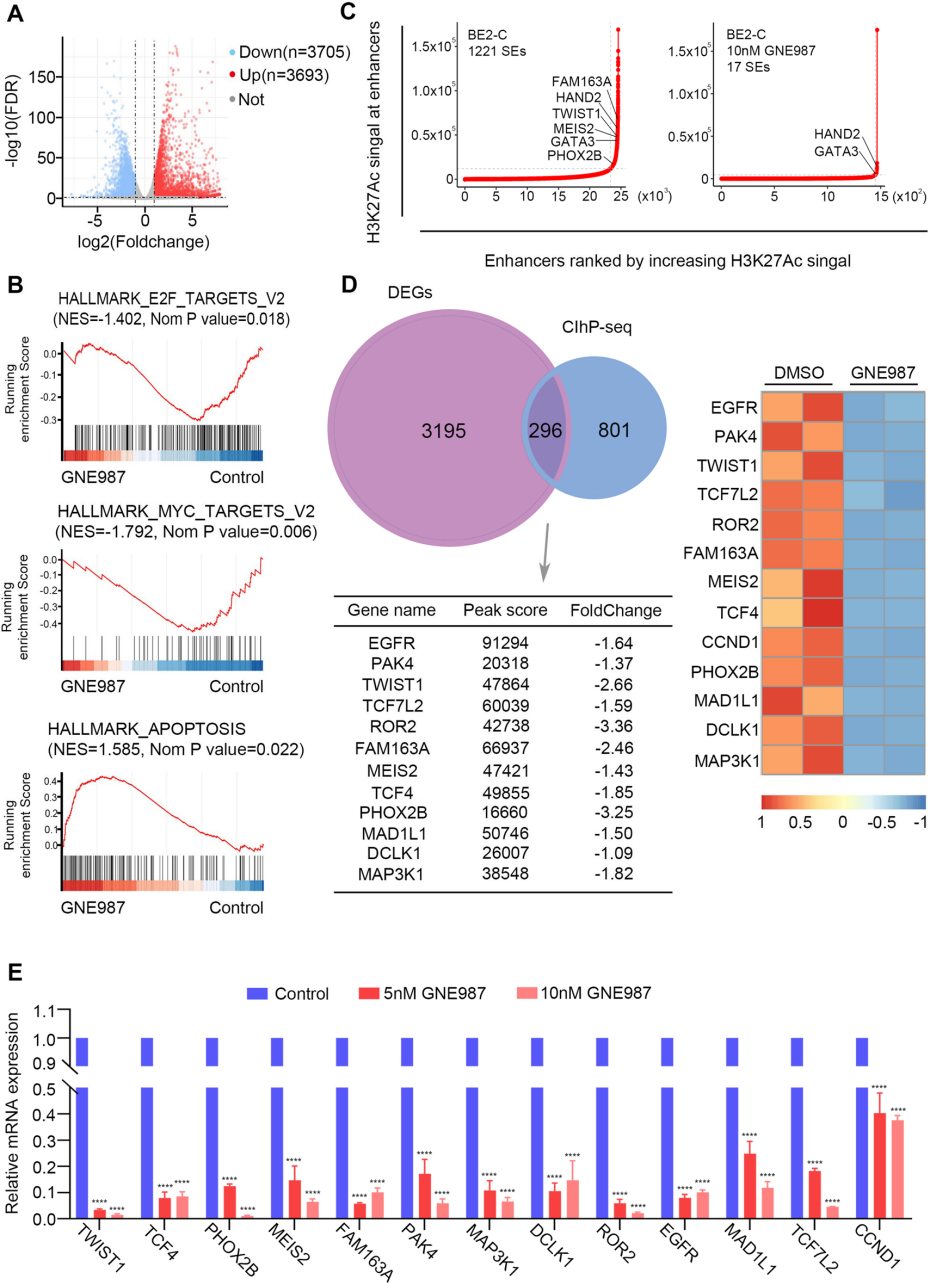
Comprehensive analysis of RNA-seq and ChIP-seq identified novel candidate oncogenic transcripts. Volcano plot of gene expression differences between the GNE987-treated and DMSO-treated SK-N-BE (2) cells. The blue and red dots highlight all the statistically significant transcripts with downregulated/upregulated expression (log2FoldChange < − 1.0 or > 1, adjusted p < 0.05). **B** Enrichment analysis results of differentially expressed genes by using GSEA Pathway Database to investigate the function of GNE987 in gene regulation. **C** Enhancers were ranked by increasing H3K27ac signal in the GNE 987 treated and nontreated SK-N-BE (2) cells. The number of SEs is shown for each group. **D** Venn diagram showing the 297 overlapping genes between the RNA-seq decreased genes and SE-related genes calculated from ChIP-seq results. The relative expression levels of the representative overlapping genes are depicted in the heatmap, of which the peak score and fold change are shown in the table. **E** RT-qPCR analysis of genes related to SE from SK-N-BE (2) treated with DMSO, 5 nM or 10 nM for 24 h. ****p < 0.0001

To determine the underlying mechanism of GNE987, we then performed RNA-seq gene expression analysis in the SK-N-BE (2) cells treated with GNE987, and mRNA expression clustering and plotted maps were used to identify differentially expressed genes. Genes with an absolute value log2FoldChange > 1 and an adjusted *p* < 0.05 were regarded as candidates. After 10 nM GNE987 treatment for 24 h, 3693 genes had upregulated expression and 3705 genes had downregulated expression in the SK-N-BE (2) cells (Fig. 7A). For investigation of the role of GNE987 in gene regulation, the functions of differentially expressed genes were annotated and enriched using HALLMARK pathway enrichment analysis. The results showed that GNE987-regulated genes were associated with the regulation of various tumour-related processes and activities, such as “HALLMARK_MYC_TARGETS_V2”, “HALLMARK_E2F_TARGETS” and “HALLMARK_APOPTOSIS” (Fig. 7B).

It is believed that GNE987 can target the BRD4-regulated transcriptional activity, destroy the structure of SEs, and block the transcriptional activation of SE-dependent oncogenes. Therefore, to identify the effects of GNE987 on SE-related genes in NB, we performed high-throughput ChIP-seq using the H3K27ac antibody to identify the changes in SE-related genes in the SK-N-BE (2) cells collected after 24 h of treatment with 10 nM GNE987. Combined with the published H3K27ac ChIP-seq data from SK-N-BE (2)-C (GSE90683), a large number of SE-associated transcripts were found to be preferentially reduced after treatment with GNE987 (Fig. 7C). Therefore, we hypothesized that the combination of SE profile analysis and gene expression analysis would allow us to identify the key oncogenes involved in the pathogenesis of NB.

Furthermore, between the RNA-seq-identified genes with downregulated expression and the SE-related genes calculated from the ChIP-seq results, 296 genes overlapped, suggesting that these GNE987-decreased genes were regulated by the corresponding SEs to participate in the development of NB (Fig. 7D). Among these overlapping genes, many have been identified as SE-associated genes, such as *PHOX2B* [42]. In addition, *EGFR* [43], *PAK4* [44], *MEIS2* [45] and *TWIST1* [46] have important roles in NB (Additional file 2: Fig. S2). Notably, some oncogenes rarely reported in NB, such as *FAM163A* and *ROR2*, belonged to the top 10% of genes that were sensitive to GNE987 treatment. To confirm these changes, the expression of SE-related genes in SK-N-BE (2) treated by DMSO or GNE987 was evaluated by real-time PCR, and the findings validated the down regulation of SE-related genes at mRNA level (Fig. 7E). These results indicated that the SE-related genes in NB were susceptible to *BRD4* degradation and that GNE987 exerts anti-cancer effects by selectively targeting these SE-related genes.

### *FAM163A* is a novel key target of GNE987

**Fig. 8:**
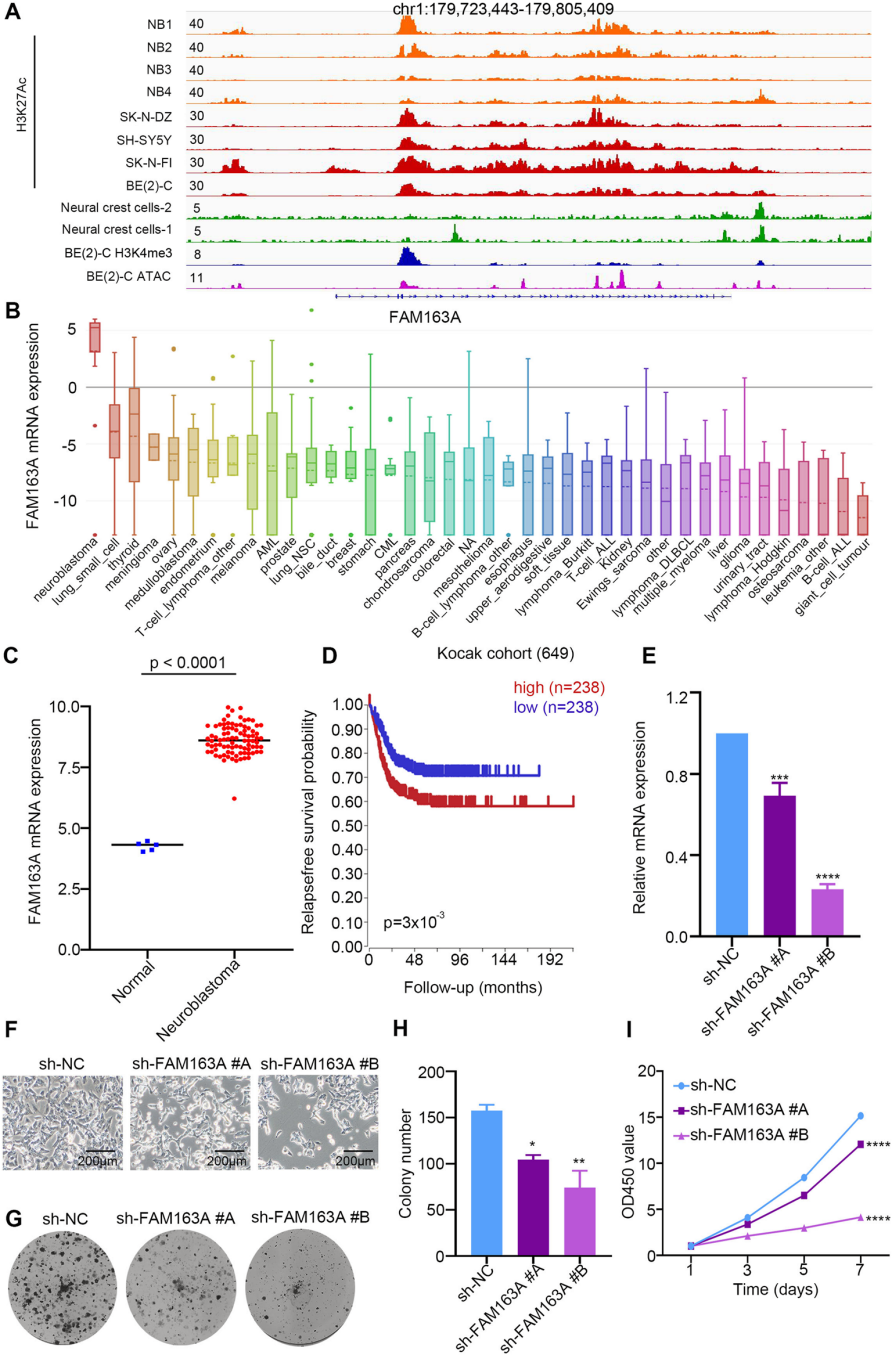
*FAM163A* is a potential oncogene of NB. **A** IGV plots showing ChIP-seq profiles of FAM163A in NB gene locus. The ChIP-seq gene tracks represent the H3K27ac signal in normal neural crest cells (green) (GSE90683), NB cell lines (red) (GSE90683) and clinical samples from NB (orange) (GSE90805). **B** *FAM163A* mRNA expression level in a broad range of tumours (generated from CCLE: https://portals.broadinstitute.org/ccle). **C** Relative mRNA expression of *FAM163A* in normal nerve cells and NB cells based on data available from the GEO database. **D** Overall survival (OS) in patients with NB with high or low levels of *FAM163A* plotted using Kaplan–Meier method (generated from R2 Genomics Analysis and Visualization Platform: r2.amc.nl). p < 0.001. **E** SK-N-BE (2) cells were transfected with sh-NC, sh-*FAM163A* #A, or sh-*FAM163A* #B and positive cells were screened using puromycin for 6 days. The mRNA expression of *FAM163A* was assessed using qRT-PCR. **F** Microscopic images of the SK-N-BE (2) cells and SK-N-BE (2) cells transfected with sh-NC, sh-*FAM163A* #A, or sh-*FAM163A* #B. **G–H** Representative images and statistical results of a clone formation assay showing the colony formation ability of the SK-N-BE (2) cells stably transfected with sh-NC, sh-*FAM163A* #A, or sh-*FAM163A* #B. *p < 0.05, **p < 0.01. **I** Proliferation rate of the SK-N-BE (2) cells transfected with sh-NC, sh-*FAM163A* #A, or sh-*FAM163A* #B. ****p < 0.0001

High H3K27Ac enrichment (indicating existing SEs) has been observed at the FAM163A lolcus in 4 NB cases and 4 NB cell lines, but not in normal neural crest cells (Fig. 8A). According to the Cancer Cell Line Encyclopedia (CCLE), with high mRNA levels in NB, *FAM163A* has a notable cancer type specificity (Fig. 8B). Thus, we further investigated *FAM163A*, which is also called NB-derived secretory protein (*NDSP*) or *C1ORF76*. Compared with *FAM163A* expression in the control group (including brain and adrenal gland tissue), *FAM163A* expression was strong and specific in tumours (Fig. 8C), identifying it as an NB marker and a predictor of high-risk NB in the clinic [47]. In addition, higher *FAM163A* mRNA levels were found to be associated with higher risk by analysing the GSE45547 dataset on the R2 platform (Fig. 8D). A previous study showed that *FAM163A*, as a specific protein actively secreted by NB, activates mitogen-activated protein kinase (*MAPK*) (*ERK1/2*) phosphorylation and promotes the malignant proliferation of NB [48].

Compared with the data from normal neural crest cells, ChIP-seq data of NB cell lines and 4 public NB samples showed substantial H3K27ac enrichment near *FAM163A*, indicating that *FAM163A* is regulated by SEs. This SE enrichment was not found in normal neural cells, indicating that *FAM163A* is specifically activated in NB cells. To verify the antitumour function of the candidate oncogene *FAM163A* in NB, we decreased its expression in SK-N-BE (2) cells using a specific shRNA (Fig. 8E). Significant induction of cell clustering, apoptosis and weakened adhesion were observed in the SK-N-BE (2) cells after *FAM163A* knockdown. There was no significant difference in growth between the sh-NC cells and the parental cells (Fig. 8F). In the clone formation assay, the sh-*FAM163A* cells produced significantly fewer colonies than the sh-NC cells (Fig. 8G and H). Consistently, CCK-8 assays revealed that downregulation of *FAM163A* expression led to decreased SK-N-BE (2) cell proliferation (Fig. 8I). Together, these results indicated that *FAM163A* sustained the growth and survival of human NB cells, which was in line with our expectations.

## Discussion

Although progress has been made in the diagnosis and therapy of NB in recent years, the prognosis of high-risk NB remains very poor [49]. The effectiveness of standard chemotherapy is hindered by drug resistance in most relapsed patients [3, 4]. In the field of biosynthesis and new drug discovery, the development of inhibitors for the transcriptional regulation of tumour cells, rather than specific genomic lesions, has provided more innovative and effective regimens. Studies have shown that many different types of cancer cells produce SEs at oncogenes, and specific SEs close to related oncogenes can help tumorigenesis [50–52]. SEs are enriched for key transcription factors, cofactors, and chromatin regulators and are superior to TEs (typical enhancers) in terms of size, transcription factor density and ability to induce transcription[53, 54]. Existing clinical studies have shown that tumour cells rely more than normal cells on a high level of SE-driven transcriptional regulation mediated by specific oncogenes, such as *RUNX1* (encoding RUNX family transcription factor 1) in AML and *MYCN* in NB [6, 55]. Thus, the synthesis of chemical inhibitors specifically targeting SEs for the clinical treatment of malignant tumours has broad application prospects [56, 57].

Currently, general SE-targeting inhibitors are usually designed for SE-related components, including *BRD4*, transcriptional *CDKs* (for example *CDK7* and *CDK9*) and the mediator complex (for example *EP300*), which are considered as core regulators of transcription. In addition to the well-studied JQ1 (a *BRD4* inhibitor), THZ1 (a *CDK7* inhibitor) and CBP30 (an *EP300* inhibitor), as new inhibitors targeting SE-related components have been tested in clinical trials and have achieved significant efficacy. For example, alvocidib (NCT03298984, NCT03969420, NCT02520011) listed by Tolero Pharmaceuticals, as a cyclin-dependent kinase inhibitor, has entered phase II trials in AML and been reported to have significant activity in patients with relapsed or refractory AML [58]. In the present study, we described an effective transcriptional disruptor, the *BRD4*-targeted inhibitor GNE987. As an important component of SEs, *BRD4* combines with RNA-Pol II and p-TEFb in the transcription of oncogenes, keeping cancer cells in a relatively immature stem cell like state and driving the occurrence and development of cancer to a certain extent [59]. Based on the relationship between high BRD4 expression and poor prognosis in NB, we inferred that it was feasible to explore GNE987 as a selective and effective cell inhibitor in NB.

Currently, selective induction of target protein degradation has emerged as a novel drug discovery strategy. PROTACs are hetero-bifunctional small molecular compounds, and are becoming a research focus because of their potential advantage over protein inhibitors in the degradation of oncoproteins in cancer treatment [25]. GNE987 is composed of a ten-methylene spacer moiety, an E3 ubiquitin ligase binding site (VHL binding fragment), and the target protein-specific ligand. BRD4 can be combined with VHL through the flexible chemical connector and be ubiquitinated for subsequent degradation by the proteasome [38]. Therefore, compared with traditional BRD4 inhibitors (such as JQ1 and BETi), GNE987 could obtain the same therapeutic effect at a lower nanomolar concentration, with greatly reduced toxicity, better binding affinity and stronger metabolic stability.

Although GNE987 has shown good efficacy in haematological malignancies, its efficacy in solid tumours is less convincing [37]. To explore the anti-NB potential of GNE987, we examined the changes in malignant characteristics in vivo and in vitro after GNE987 treatment. NB has a strong capacity for malignant proliferation. Our study showed that GNE987 inhibited the proliferation of NB cells in a time- and dose-dependent manner and induced cell cycle arrest and apoptosis. Moreover, knocking down *VHL* reduced the inhibitory efficiency of GNE987 on NB cells. Accordingly, VHL overexpression increased the sensitivity of NB cells to GNE987. In addition, in the NB mouse model, compared with the controls, GNE987 effectively inhibited the tumour growth of nude mice, without any serious side effects or pathological changes to important organs. In summary, our results showed that GNE987 had strong antitumour activity against NB in vivo and in vitro, which may have clinical value for the treatment of patients with NB.

To clarify the potential mechanism, we used RNA-seq to detect the changes in NB total transcripts after treatment with GNE987 for 24 h and identified differentially expressed genes that were closely related to critical cancer-associated features, such as “HALLMARK_MYC_TARGETS_V2”, “HALLMARK_APOPTOSIS” and “HALLMARK_P53_PATHWAY”. Previous studies have shown that SEs are involved in driving the hyperactivation of transcriptional regulators. We further explored the effect of GNE987 on SE-related oncogenes in NB and tested our hypothesis that SEs could be accurately located on previously uncharacterized oncogenes. Thus, we identified SE-related oncogenes by comprehensively considering the ChIP-seq and RNA-seq datasets. Interestingly, in addition to a large number of well-defined oncogenes in NB, such as "*MEIS2*", "*TWIST1*", *“PHOX2B”* and "*GATA3*", there were also a number of transcripts rarely reported in NB. To narrow the candidate list, we performed multistandard feature selection of candidate oncogenes in NB. Subsequently, after preliminary screening using overall survival analysis and a series of functional experiments, we identified reported oncogenes that were important for the malignant NB phenotype, *FAM163A*. According to previous studies, *FAM163A*, which is also called NB-derived secretory protein (*NDSP*) or *C1OEF76*, has tumour specificity in NB and activates the extracellular regulated kinase (*ERK*) signalling pathway by combining with multiple 14-3-3 family members to promote the proliferation of tumours [47, 48, 60]. Here, we showed that *FAM163A* is an oncogene associated with SEs that is highly active in NB. The present study demonstrated that knockdown of the FAM163A gene had an inhibitory effect on proliferation and induced apoptosis in NB cells.

In conclusion, we showed that GNE987 exerts prominent antitumour effects by selectively blocking BRD4 and SE-regulated oncogene transcription, and inducing strong cell apoptosis and cell cycle arrest. Additionally, we used combinatorial methods to identify highly activated and interesting oncogenes in NB cells. This strategy will be valuable as a reference for other cancers with high levels of genetic abnormalities. However, since we only detected the phenotype of cell proliferation, candidate genes for other cell functions such as tissue invasion, metastasis, and sustained angiogenesis remain to be identified.

## Methods

### Cell culture

The NB cell lines [SK-N-BE (2), IMR-32, SK-N-SH and SH-SY5Y] and 293FT were cultured in DMEM or MEM medium (Biological Industries, Israel), supplemented with 10% heat-inactivated FBS (Dongling Biotech, Soochow, China), 1% penicillin–streptomycin (Millipore Sigma, Darmstadt, Germany) and 0.1% Ciprofloxacin (Heowns Biochem Technologies, Inc., Tianjin, China) in a humidified incubator with 5% CO_2_ at 37 °C, and were routinely assessed for mycoplasma contamination. All the cell lines were purchased from the cell bank of the Chinese Academy of Sciences within the last 5 years, and verified by short tandem repeat analysis between 2018 and 2019.

### Tissue microarray and immunohistochemistry

The tissue microarray containing 27 samples of NB patients and 5 peripheral nerve tissues was purchased from Biomax (MC642, Derwood, USA). NB xenograft tumor tissues and mouse organ tissues obtained from animal experiments were fixed in 4% paraformaldehyde (Beyotime, Shanghai, China) and embedded in paraffin. Sections were deparaffinized in xylene (MACKLIN, Shanghai, China), rehydrated in graded alcohols (Sinopharm, Beijing, China), and washed in distilled water. Antigens were retrieved by boiling the sections in citric acid-based antigen unmasking solution (Thermo Fisher, Waltham, MA, US) for 30 min, then incubated with primary anti-*BRD4* (1:200, ab128874, Abcam, Cambridge, UK) or Ki-67 (1:200, ab15580, Abcam, Cambridge, UK) overnight at 4 °C followed by rabbit specific HRP/DAB detection kit (Cat: ab64261, Abcam, Cambridge, UK) and hematoxylin (Beyotime, Shanghai, China) in compliance with protocols. Staining results were independently evaluated by two experienced pathologists. The total scoring (TS) results were scored by multiplying the percentage of positive cells (P) by the intensity (I). Formula: TS = P × I.

### Cell proliferation and viability assays

NB cell lines [SK-N-BE (2), IMR-32, SK-N-SH and SH-SY5Y] 1 × 10^4^ per 200 μl were seeded into 96-well plates and incubated overnight at 37 °C. Subsequently, cells were treated with gradient concentrations (from 0.5 to 5 × 10^3^ nM) of GNE987 (MedChemExpress, NJ, USA) and incubated for another 24 h. The control group was treated with equal volume of DMSO (final DMSO volume, < 0.1% cell culture medium (v/v) and toxicity < 0.1%). The half-maximal inhibitory concentration (IC50) of GNE987 was measured with CCK-8 assay (Dojindo Molecular Technologies, MD, USA) as previously described [34]. IC50 values and relative survival rates of NB cells treated with GNE987 were calculated using GraphPad Prism8.4.3 (GraphPad Software, CA, USA). Treatment with each drug concentration was replicated three times and the background reading of the medium from each well was subtracted to standardize the results.

### Cell cycle analysis

Cell cycle analysis was performed as previously described [34]. NB cells were seeded into six-well plates to adhere overnight and treated with GNE987 at the indicated concentrations for another 24 h. The cells were collected after being washed by cold PBS, then fixed in 70% ethanol overnight and punched by 0.5% TritonX-100. Then the samples were treated with 25 µg/ml RNase A (#7013, CST, MA, USA) and 1.5 µmol/l propidium iodide (PI) (P4170, Sigma, Darmstadt, Germany) following the manufacturer’s instructions. Finally, samples were detected by Beckman Gallios™ Flow Cytometer (Immunotech Beckman Coulter, CA, USA) and analyzed using MultiCycle AV DNA analysis software (version 328, Verity Software House).

### Cell apoptosis assay

Cell apoptosis analysis was performed as previously described [61]. NB cells were treated with GNE987 at the predetermined concentrations in six-well plates at 37 °C. Following 24 h incubation, cells were collected and stained with AnnexinV-FITC and PI following the instructions of the FITC-Annexin V apoptosis detection kit (556420, BD Biosciences, NJ, USA). Cell apoptosis was analyzed using flow cytometry as aforementioned.

### Quantitative real-time polymerase chain reaction

RT-qPCR was performed as described previously. Total RNA from NB cells was isolated by RNeasy Mini kit (74104, Qiagen GmbH, MD, Germany) according to the manufacturer’s protocol. Reverse transcription was carried out using 200 U M-MLV reverse transcriptase (Promega Corporation, WI, USA), 20 U RNase inhibitor (Thermo Fisher, Waltham, MA, US), and 500 ng random primers (Promega Corporation, WI, USA) on an ABI PCR platform (Applied Biosystems, Thermo Fisher, Waltham, MA, US). The two-step PCR procedure was as follows: initial denaturation at 70 °C for 5 min, denaturation for 60 min at 37 °C and annealing/extension for 10 min at 85 °C.

The qPCR amplification was performed in a LightCycler® 480 (Roche, Mannheim, Germany) using universal thermal cycling parameters (an initial 95 °C for 10 min and 45 cycles of 15 s at 95 °C, 15 s at 60 °C, and 60 s at 72 °C. After that, the melting curve for 10 s at 95 °C, 60 s at 65 °C and 30 min at 40 °C). Target gene was amplified using specific oligonucleotide primer and human *GAPDH* gene was used as an endogenous control. Expression quantification of target gene was calculated with the 2–ΔΔCT method. The primer sequences are as following: FAM163A, 5′GCTCCCACATACTACAAAGAGGG-3′ (Forward) and 5′-CCAGGTCACCGGGTAACTC-3′ (Reverse), GAPDH, 5′ATCATCCCTGCCTCTACTGG-3′ (Forward) and 5′CCCTCCGACGCCTGCTTCAC-3′ (Reverse).

### Western blot analysis

NB cells were collected and lysed in RIPA buffer (Beyotime, Shanghai, China) containing complete protease inhibitor cocktail (Sigma, Darmstadt, Germany) to obtain total protein. Western blotting was performed as described previously [61] using the following primary antibodies: anti-Caspase-3 (9661S, CST, MA, USA), poly (ADP-ribose) polymerase 1 (PARP, 9542S, CST, MA, USA), GAPDH (AP0063, 1:5,000, Bioworld Technology Inc.), Cyclin D1 (2978S, CST, MA, USA), BRD2 (5848S, CST, MA, USA), BRD3 (11859-1-AP, Proteintech, IL, USA), BRD4 (13440S, CST, MA, USA), N-Myc (9405S, CST, MA, USA), C-Myc (9402S, CST, MA, USA), VHL (68547S, CST, MA, USA) and incubated overnight at 4 °C. The following day, after incubating with goat anti-rabbit (111-035-003) or anti-mouse IgG (H + L; 115035-003) HRP-conjugated secondary antibodies (both 1:3000; both Jackson ImmunoResearch Laboratories, PA, USA) for 1 h, membranes were developed using an ECL ultra-sensitive luminescent fluid (Thermo Fisher Waltham, MA, US) using LAS 4010 (GE Healthcare Life Sciences, Little Chalfont, UK Cytiva) imaging system and visualized using the ImageQuant TL 8.1 software. The loading control was *GAPDH* for all blots. ImageJ software was used for band quantification.

To determine the role of proteasome, MG132 (Sigma, Darmstadt, Germany) was used to inhibit the activity of the proteasome activity. After treatment with 5 nM GNE987 and series concentrations of MG132 for 24 h, NB cells were collected and BRD2, BRD3 and BRD4 protein were determined by Western blot.

### Xenograft preparation and GNE987 treatment in nude mice

For the NB in vivo tumor model, 2 × 10^6^ luciferase-labeled SK-N-BE (2) cells were xeno-transplanting hypodermically into the armpit of 3–4 week-old nude mice purchased from Cavens Biogle Model Animal Research Co.Ltd. NB tumor model mice were average randomly assigned to Ctrl or GNE987 treatment groups. Following initial tumor establishment, bioluminescence (BLI) was conducted weekly by BERTHOLD (Germany) in vivo imaging system (LB987). Tumor-bearing mice were injected 100 μl 0.25 mg/kg GNE987 or the vehicle of the same dosages (5% Kolliphor^®^HS15) as the test group every two days when the tumor BLI flux reached the range of 3–5 × 10^5^ photons/second. Tumors volume and weight of mice were measured every 2–3 days. Tumors in both groups of mice were harvested when the tumor size of Ctrl group exceeded 1000 mm^3^, which was defined as the survival endpoint. All animal procedures in this study were approved and licensed by the Animal Care and Use Committee at Children’s Hospital of Soochow University.

### RNA-sequencing (RNA-seq) and data analysis

RNA-seq was implemented using the protocols provided by Novogene (Novogene Co., Ltd., Beijing, China). Total RNA was isolated using Trizol reagent (Invitrogen Thermo Fisher, Waltham, MA, US). RNA purification, library construction and sequencing were performed by Novogne. Gene expression profiles in SK-N-BE (2) cells treated with 10 nM GNE987 or the same volume DMSO for 24 h were identify by RNA-seq. RNA-seq original data have been submitted to the Gene Expression Omnibus (GEO) database (GSE190002). The Bioconductor DESeq2 package 1.32.0 was used to identify differentially expressed genes. Gene set enrichment analysis (GSEA) were performed using the clusterProfiler package 4.0.5 in R 4.1.1.

### Chromatin immunoprecipitation (ChIP-seq) and data analysis

The ChIP assay was performed using established protocols as previously described [62]. SK-N-BE (2) cells were seeded into T75 and divided into two groups, the treatment group was treated with 10 nM of GNE987 for 24 h, while the control group was treated with the same volume of solvent for the same time. After washing by PBS twice, the same amount of cells in both groups were fixed 1% formaldehyde for 10 min at room temperature under gently shaking. Then, the cross-linking was quenched by 0.125 M glycine for 5 min. The cells collected after centrifugation at 200×*g* for 5 min were lysed in icy cell lysis buffer (0.01 mM NaCl, 0.5 M EDTA pH 7.5, 1 M Tris pH 7.5, 0.2% NP-40) containing protease inhibitor cocktail (Sigma, Darmstadt, Germany). After that, cells were ruptured by gently blowing with a 1 ml insulin needle (this step is repeated twice). The precipitate obtained by centrifugation at 13,800×*g* for 5 min was suspended in the shearing buffer (20% SDS, 0.5 M EDTA pH 8.0, 1 M Tris pH 8.0) containing protease inhibitor cocktail, and sonicated using ultrasonic cell disruptor (M220, Covaris, Massachusetts, USA) for 5 min at 4 °C to shear genomic DNA into 300–800 bp fragments in length. The sonicated samples were centrifuged at 13,800×*g* for 5 min at 4 °C. A 20 μl aliquot from the supernatant was stored at − 20 °C and used as input for each sample. The rest samples were pre-combined by incubating with a H3K27ac antibody (ab4729, Abcam) at 4 °C overnight. The next day, Dynabeads Protein G beads (10004D, Thermo Fisher Scientific) were added into the sample for immunoprecipitation reaction at 4 °C for 4 h. The antibody-chromatin complex with beads was washed six times with lysis buffer, and washed twice with TE buffer (Sigma). Afterward, the anti-body-chromatin complexes were eluted from the Protein A/G beads in EB buffer (1 M NaHCO_3_, 20% SDS) and subjected to reverse crosslinking by incubating with 5 M NaCl at 65 °C overnight. 10 mg/ml RNase (#7013, CST) was added and incubated at 37 °C for 30 min to remove contaminating RNAs. 20 mg/ml Proteinase K (AM2546, Invitrogen), 0.5 M EDTA (pH 8.0) and 1 M Tris HCl (pH 8.0) were added and incubated at 45 °C for 1 h to remove proteins. The remaining DNA fragments were purified by PCR Purification Kit (250) (QIAGEN) and gel electrophoresis were performed to detect the size of DNA fragments. ChIP-seq original data have been submitted to the Gene Expression Omnibus (GEO) database (GSE194430).

### Vector construction and infection

Envelop plasmid and packaging plasmid (pMD2.G, 12259; psPAX2, 12260) were provided from Addgene (MA, USA). Multiple shRNA sequences targeting VHL, FAM163A and negative control in the *PLKO.1* lentiviral vector was designed and constructed by IGEbio (Guangzhou, China). The sequences of shRNA targeting VHL and FAM163A are listed. Overexpression sequence targeting *VHL* in PLVX-Flag lentiviral vector and negative control (PLVX-NC) were purchased from IGEbio. The transfections were performed according to the protocol provided by the manufacturer (PEI, Polysciences, PA, USA). The indicated cells infected with the lentiviral vectors were cultured with 10 μg/ml puromycin (Invitrogen, Thermo Fisher, Waltham, MA, US) for a week to establish the stably transfected NB cells. The targeting sequence for *VHL* was 5′-CCGGGCTCAACTTCGACGGCGAGCCCTCGAGGGCTCGCCGTCGAAGTTGAGCTTTTTGAATT-3′; the targeting sequence for *FAM163A* #A was 5′-CCGGGCACGACCTTCCCACGCATCCCTCGAGGGATGCGTGGGAAGGTCGTGCTTTTTGAATT-3′; the targeting sequencing for *FAM163A* #B was 5′-CCGGGAGGCCTTCACCAATCCAAGGCTCGAGCCTTGGATTGGTGAAGGCCTCTTTTTGAATT-3′.

### Statistical analysis

All experiments were independently performed in triplicate at least three times. Statistical analyses were performed by GraphPad Prism 8.3.0 (GraphPad Software, Inc., San Diego, CA, USA). Differences between the two groups were calculated using a 2-tailed paired *Student’t* test. Differences between multiple groups were compared using one-way ANOVA. *P* values less than 0.05 were regarded as statistically significant (*p < 0.05, **p < 0.01, ***p < 0.001, ****p < 0.0001). Means ± Standard Deviation (SD) are shown.

## Supplementary Information

**Additional file 1: Figure S1.** PI-labeled cell cycle of NB cells were analyzed after treatment with DMSO or different concentrations of GNE987 for 24 h.

**Additional file 2: Figure S2.** IGV plots showing ChIP-seq profiles of the indicated factors in the SK-N-BE (2) gene locus. The ChIP-seq gene tracks represent the H3K27ac signal in normal neural crest cells (green) (GSE90683), NB cell lines (red) (GSE90683) and clinical samples from NB (orange) (GSE90805).

## Data Availability

The datasets used and/or analyzed during the current study are available from the corresponding author on reasonable request. RNA-seq and original data have been submitted to the GEO database with Accession Number GSE190002.

## Abbreviations

NB: Neuroblastoma
SE: Super-enhancer
MYCN: Encoding MYCN proto-oncogene, BHLH transcription factor
MYC: Encoding MYC proto-oncogene, BHLH transcription factor
RNA-seq: RNA-sequencing
ChIP-seq: Chromatin immunoprecipitation
