# Supplementary figures and images for: BRD4 inhibitor GNE987 exerts anti-cancer effects by targeting super-enhancers in neuroblastoma
Source: Cell Biosci. 2022 Mar 18;12:33. doi: 10.1186/s13578-022-00769-8 (PMC8932231; doi:10.1186/s13578-022-00769-8)

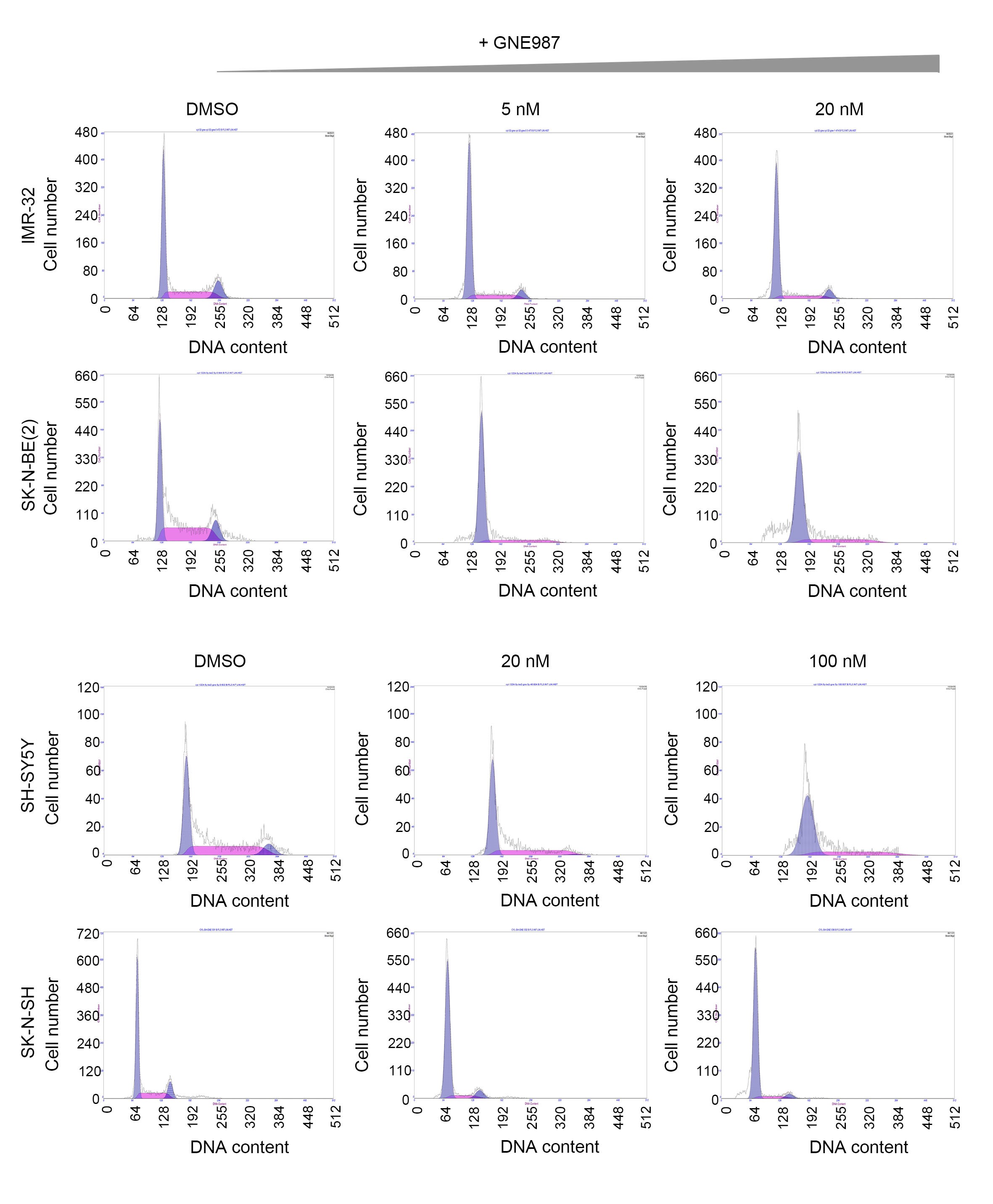

Supplement: Supplementary file 1 — Additional file 1: Figure S1. PI-labeled cell cycle of NB cells were analyzed after treatment with DMSO or different concentrations of GNE987 for 24 h. [file 13578_2022_769_MOESM1_ESM.tif]

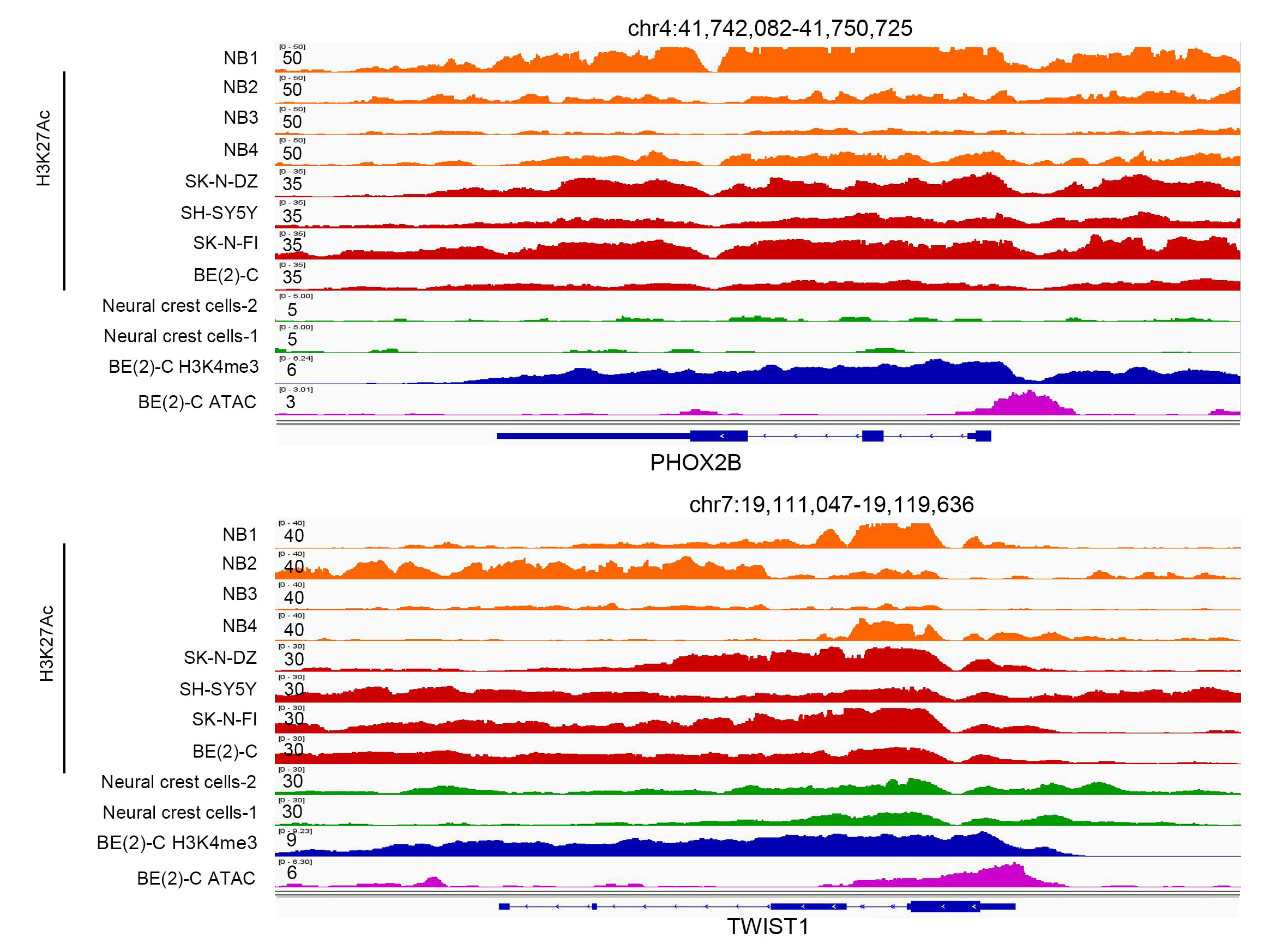

Supplement: Supplementary file 2 — Additional file 2: Figure S2. IGV plots showing ChIP-seq profiles of the indicated factors in the SK-N-BE (2) gene locus. The ChIP-seq gene tracks represent the H3K27ac signal in normal neural crest cells (green) (GSE90683), NB cell lines (red) (GSE90683) and clinical samples from NB (orange) (GSE90805). [file 13578_2022_769_MOESM2_ESM.tif]
